# Clinical and prognostic characteristics of 95 cases of Langerhans cell histiocytosis in children: a single-institute experience from 2013 to 2020

**DOI:** 10.1080/07853890.2021.1966085

**Published:** 2021-08-30

**Authors:** Xue Tang, Ju Gao, Zhi-gui Ma, Xia Guo, Qiang Li, Zhi Wan, Jing-jing Sun

**Affiliations:** a Department of Pediatrics, West China Second University Hospital, Sichuan University, Chengdu, China; b Key Laboratory of Birth Defects and Related Diseases of Women and Children, Sichuan University, Ministry of Education, Chengdu, China

**Keywords:** Langerhans cell histiocytosis, clinical characteristics, therapy, prognosis, children

## Abstract

**Background:**

This study aimed to understand the clinical characteristics and outcomes of children with Langerhans cell histiocytosis (LCH) in China.

**Methods:**

We conducted a retrospective study of 95 paediatric patients with LCH in West China Second University Hospital of Sichuan University between July 2013 and August 2020.

**Results:**

The onset age of multisystem LCH (MS-LCH) patients with risk organ (RO) involvement was younger than that of MS-LCH without RO involvement (*p* = .002) and single system LCH (*p* < .001) patients; bone was the most frequently involved organ, followed by the skin. Of all, the *BRAF-V600E* mutation was detected in 48 out of 84 patients who underwent gene analysis. Additionally, in our study, *BRAF p.N486_T491 > K*, *BRAF p.L485_P490delinsF*, *BRAF p.R506_K507insLLR*, *ARAF p.Q349_F351delinsL* and *MAP2K1 p.Q58_E62del* were known mutations in the mitogen-activated protein kinase (*MAPK*) pathway. The *BRAF-V600E* genotype in the tissue and plasma prior to therapy were detected in 16 patients, and the concordance was only 37.5% (6/16). According to the modified LCH-III-based-protocol, JLSG-02 protocol chemotherapy, and vemurafenib, the estimated five-year overall survival, event-free survival (EFS) and cumulative reactivation rates of 95 patients were 98.8%, 74.6% and 24.5%, respectively. The EFS rate in good responders was better than that in poor responders at 12-week (HR = 0.022, 95%CI 0.002–0.231, *p* = .002), and EFS was not affected by age, RO involvement or *BRAF-V600E* mutation. Regarding sequelae, nine patients had central diabetes insipidus and two had growth retardation.

**Conclusions:**

In this study, LCH was a highly heterogeneous disease characterized molecularly by MAPK-pathway activating mutations. Vincristine, prednisone and cytarabine-based chemotherapy combined with vemurafenib improved the prognosis of childhood LCH. In future, prospective clinical trials and novel therapeutic strategies should be developed to improve outcomes in paediatric patients with LCH.

**KEY MESSAGE**
- Children with Langerhans cell histiocytosis in China present highly heterogeneous clinical characteristics, with up to 60% of cases harbouring mutations in *MAPK* pathway.
- Treatment response at 12-week is associated with EFS in our study.
- Vincristine, prednisone and cytarabine-based chemotherapy combined with vemurafenib improved the prognosis of Chinese childhood LCH, but the reactivation rate is still high.

## Introduction

Langerhans cell histiocytosis (LCH) is the most common histiocytic disorder in children, with a prevalence of approximately 5–9 cases per 1 million children under the age of 15 years [1,2]. Recently, LCH has been defined as a disorder driven by misguided myeloid differentiation [3] and identified with mutually exclusive somatic mutations in mitogen-activated protein kinase (MAPK) pathway genes in approximately 75% of LCH cases [4]. The extent of the disease or organs and systems involved form the basis of the LCH clinical classification system: single-system LCH (SS-LCH) with one organ/system involved (uni- or multifocal SS-LCH) and multisystem LCH (MS-LCH) with two or more organs/systems involved, stratified by risk organ (RO) involvement (high (RO+) or low (RO–) risk groups) [5]. The single site SS-LCH usually requires local therapy or observation, while the current standard of frontline treatment in multifocal LCH is empirically derived chemotherapy [5–7].

In LCH-III trial, the five-year survival rates of children with RO– MS-LCH and RO + MS-LCH were improved to 99% and 84%, respectively. Moreover, the LCH-III trial revealed that treatment prolongation can reduce reactivation in the RO– MS-LCH group, but methotrexate in the initial therapy did not improve the outcome of the RO + MS-LCH group [5]. The LCH refractory to the standard vinblastine and steroid regimen has a poor survival, which is improved by highly toxic second-line chemotherapy [8] or haematopoietic stem-cell transplantation (HSCT) [9]. However, a moderate dose of cytarabine monotherapy shows promising results in refractory LCH with a less aggressive approach [10]. Vemurafenib is also safe and effective in children with refractory *BRAF-V600E*-positive LCH, but the disease is always reactivated with withdrawal of vemurafenib [11].

To date, few English studies have reported on the treatment and prognosis of Chinese children with LCH. In our hospital, we adopted a modified LCH-III-based-protocol chemotherapy as first-line therapy, JLSG-02-protocol chemotherapy and vemurafenib as salvage therapy, and obtained satisfactory results. Herein, we performed a retrospective study to report the clinical characteristics and outcomes of 95 LCH cases in our institution over the past seven years to gain further insight into children with LCH in China.

## Materials and methods

### Study population

We reviewed all children diagnosed with LCH between July 2013 and August 2020 from the West China Second University Hospital of Sichuan University. A total of 118 patients were identified. The three exclusion criteria were: (1) the diagnosis of LCH was not established by biopsy-proven with typical pathology and positive CD1a, and/or Langerin staining. (2) Patients who were not treated at our hospital after the initial diagnosis of LCH. (3) Patients who were lost to follow-up. Finally, 95 patients were selected for this retrospective study. This study was approved by the Ethics Committee of the West China Second University Hospital of Sichuan University (No. 162). The need for informed consent was waived off as data were anonymized.

### Clinical information

We collected clinical information data of 95 patients on age, sex, involved organs, gene analysis, initial treatment response and complications of disease after treatment from medical records. The liver, spleen and haematopoietic system were defined as ROs [5]. The *BRAF-V600E* mutation in lesions was detected using real-time fluorescent quantitative polymerase chain reaction (PCR) assay. Next-generation sequencing was used to identify mutations in MAPK pathways in lesions without the *BRAF-V600E* mutation. Quantitative detection of the circulating cell-free (ccf) *BRAF-V600E* mutation was performed using a droplet-digital PCR assay. The ccf *BRAF-V600E* load less than 0.05% was defined as low level.

### Treatment protocols

All MS-LCH patients received systemic chemotherapy, while the SS-LCH patients had varied treatment protocols. Systemic chemotherapy was administered to multifocal SS-LCH and special site disease (including vertebrae, craniofacial bone, eye, ear, oral cavity and central nervous system). The SS-LCH patients with involvement of other sites could choose curettage, systemic chemotherapy or observation depending on the patients’ characteristics and their parents’ choice.

**Figure 1.**
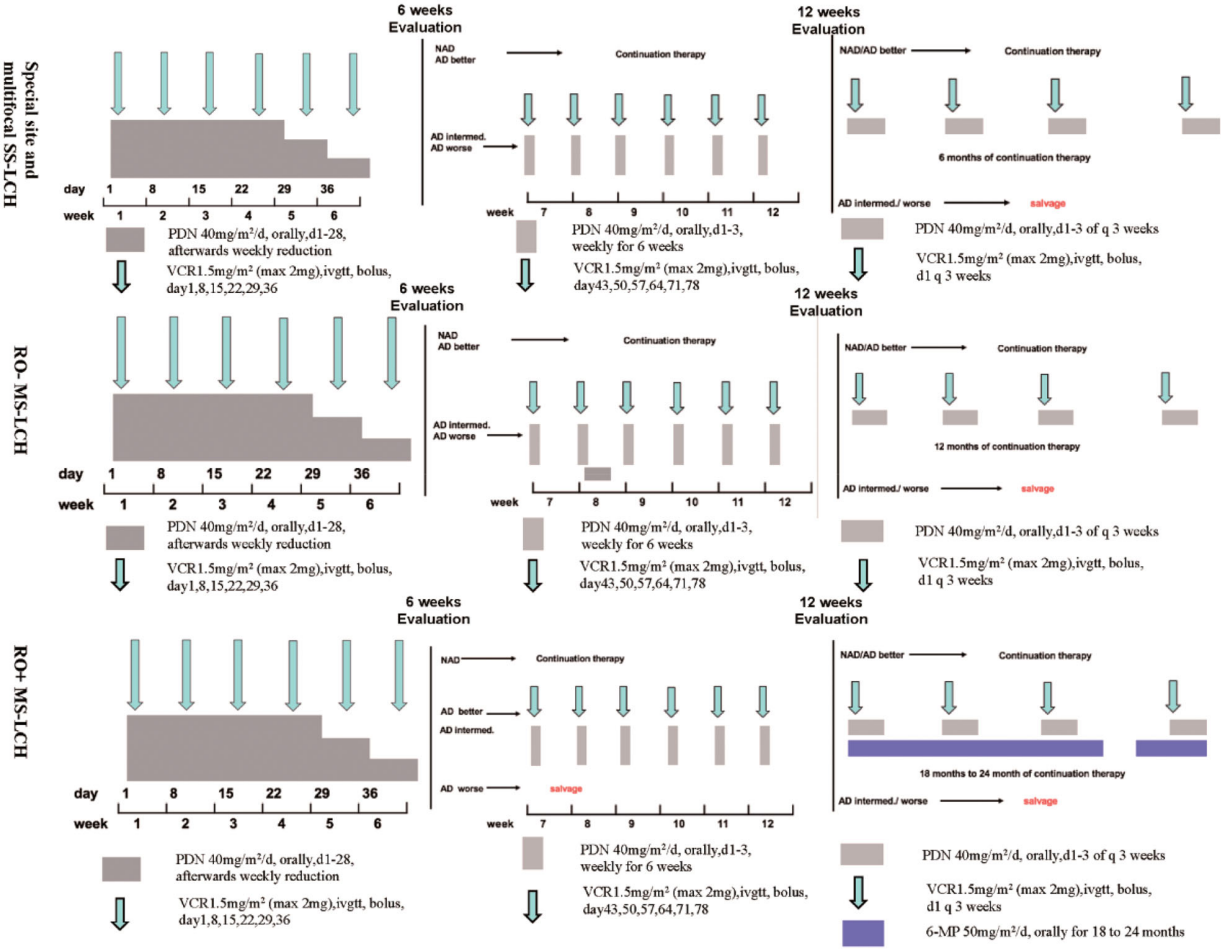
Treatment protocol of modified LCH-III-based chemotherapy. PDN: prednisolone; VCR: vincristine; 6-MP: 6-mercaptopurine; NAD: non-active disease; AD: active disease; intermed.: intermediate.

The first-line chemotherapy protocol in our institution was a modified-LCH-III based protocol (Figure 1) [5]. Four alterations were made in modified-LCH-III based protocol compared with LCH-III protocol. First, continuation therapy time was prolonged for MS-LCH, receiving a 12-month continuation treatment (prednisone, vincristine) in RO– groups and an 18- to 24-month continuation treatment in RO + groups (prednisone, vincristine, 6-mercaptopurine). Second, multifocal and special site SS-LCH patients were also administered to modified-LCH-III based protocol chemotherapy, accepting a 6- or 12-week induction therapy (prednisone, vincristine) followed by a six-month continuation treatment (prednisone, vincristine). Third, vincristine was used to replace vinblastine in modified-LCH-III based protocol due to lack of vinblastine in our hospital. Finally, because LCH-III clinical trial revealed methotrexate added no benefit, methotrexate was removed in modified-LCH-III based protocol.

**Figure 2.**
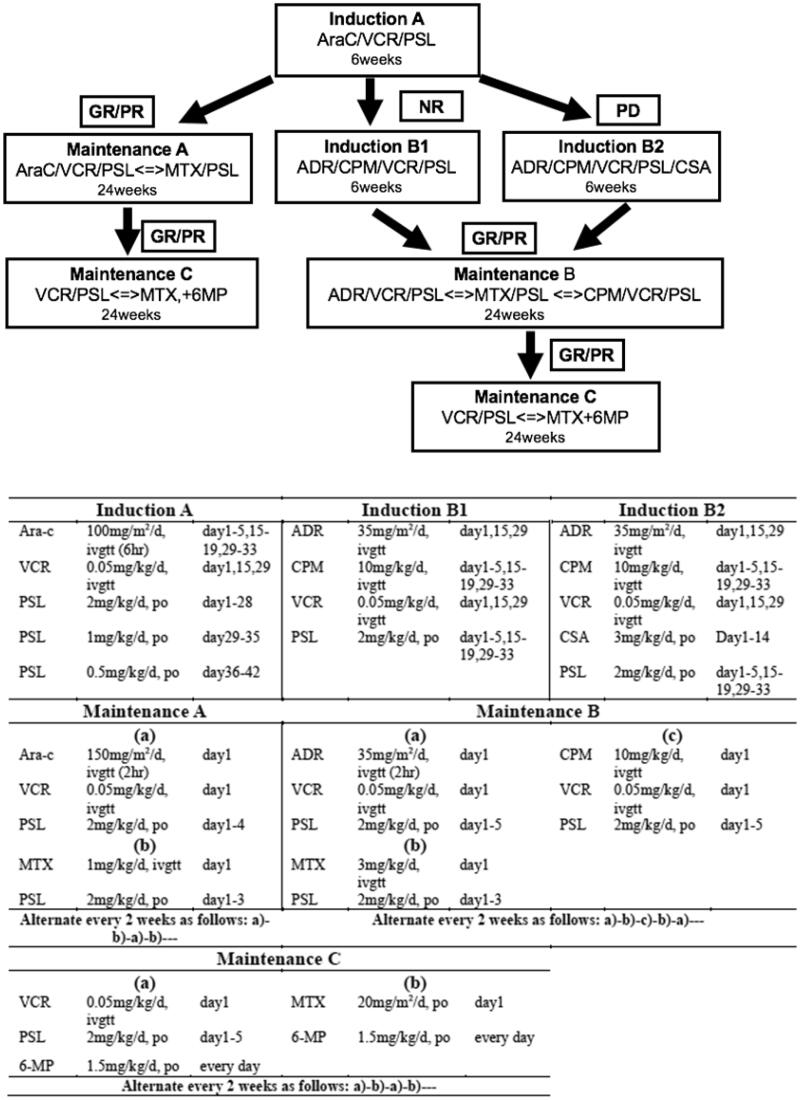
Treatment plans of JLSG-02 protocol chemotherapy. Ara-C: cytarabine; VCR: vincristine; PSL: prednisolone; MTX methotrexate; 6-MP: 6-mercaptopurine; ADR: adriamycin; CPM: cyclophosphamide; CSA: cyclosporine A; GR: good response; PR: partial response; NR: non-response; PD: progressive disease.

Patients with reactivation of LCH adopted different treatment strategies. When the reactivation developed after the withdrawal of chemotherapy, we selected the modified LCH-III-based protocol chemotherapy again. However, if reactivation patients failed to receive prednisone and vincristine, second-line therapy was selected. Additionally, when the reactivation developed during modified-LCH-III-based protocol chemotherapy, we directly switched to second-line therapy. JLSG-02 protocol chemotherapy (Figure 2) [6] and vemurafenib (20 mg/kg/d, bid) were the second-line therapies in our hospital.

Before July 2019, JLSG-02 protocol chemotherapy was selected to all the patients who were refractory prednisone and vincristine. From July 2019, vemurafenib was used to salvage LCH parallel with chemotherapy in our hospital. Patients with *BRAF-V600E* mutation refractory to prednisolone and vinblastine were candidates for vemurafenib therapy. After the parents signed informed consent form, the patients would oral vemurafenib daily.

Therapy for patients with reactivation more than once were different. If patients were sensitive to prednisone and vincristine, modified-LCH-III-based protocol chemotherapy was still adopted. If patients were refractory to prednisone and vincristine, but sensitive toJLSG-02 protocol chemotherapy, JLSG-02 protocol chemotherapy alone or with vemurafenib was selected. If patients were refractory to JLSG-02 protocol chemotherapy, vemurafenib was added to JLSG-02 protocol chemotherapy. If patients were refractory to JLSG-02 protocol chemotherapy and vemurafenib, patients were advised to transfer to other medical centre for HSCT or participating cladribine or cladribine clinical trials.

### Outcomes

The primary outcome measures were overall survival (OS) and event-free survival (EFS). OS was defined as the time between the initiation of diagnosis and death from any cause, while EFS was defined as the time between the initiation of diagnosis and the first event. Disease reactivation, secondary malignancy and death from any cause were defined as events. For patients who did not experience any event, OS and EFS were defined as the time to the last follow-up (31 August 2020).

Other studied outcomes were initial response rate, cumulative activation rate and sequelae. The initial treatment response was evaluated at the end of induction therapy (at 6 and 12 weeks). The definition of response to treatment was assessed by the International LCH Study Group criteria [5], including non-active disease (NAD), active disease (AD)/better, AD/intermediate and AD/worse. The NAD and AD/better were defined as good responses, and AD/intermediate and AD/worse were regarded as poor responses. The cut-off date of analysis was 31 August 2020, and the median follow-up was 25 months (range, 1–85 months).

### Statistical analysis

Statistical analysis was performed using SPSS version 22 (IBM Corporation, Armonk, NY). Continuous variables were presented as median values and ranges. The Mann–Whitney *U* test was used to compare differences in the continuous variables between subgroups. Time-to-event data were analysed using the Kaplan–Meier method and compared using the log-rank test. Statistical significance was set at *p* < .05. Hazard ratios and the corresponding confidence intervals (CIs) were calculated using a Cox proportional hazards model.

## Results

### Patient characteristics

A total of 95 patients with LCH were studied, including 20 with RO + MS-LCH, 24 with RO − MS-LCH, 50 with SS-LCH and one with mixed LCH and Erdheim-Chester disease (ECD). Their median age was 28 months (range, 2–164 months). The onset age of the RO + MS-LCH patients (median 9 months, range 3–52 months) was younger than that of RO– MS-LCH (median 30 months, range 2–164 months, *p* = .002) or SS-LCH (median 40 months, range 7–163 months, *p* < .001). At diagnosis, the bone was the most frequently involved organ (76/95, 80%), followed by skin (23/95, 24%), lymph nodes (18/95, 19%), lungs (17/95, 18%), liver (17/95, 18%), spleen (9/95, 9%), haematopoietic system (9/95, 9%), ear (9/95, 9%) and pituitary (9/95, 9%). Additionally, involvement of the kidney, palate, thymus, gums, brain, spinal cord, nasal sinus, oesophagus and mediastinum was rare in our patients. The ethnic distribution of the 95 patients in our study was as follows: Han-nationality, 89; Zang-nationality, 2; Tujia-nationality, 2; Yi-nationality, 1; and Bai-nationality, 1. The clinical characteristics of the 95 patients are summarized in Table 1.

**Table 1.** Patients’^a^ demographics and characteristics.

Patients	MS-LCH (RO+)	MS-LCH (RO−)	SS-LCH
	20	24	50
Gender			
Male	8 (40%)	12 (50%)	29 (58%)
Female	12 (60%)	12 (50%)	21 (42%)
Age of onset (months)			
Median	9	30	40
Minimum	3	2	7
Maximum	52	164	163
Younger than 2 years	17 (85%)	8 (33%)	14 (28%)
Nationality			
Han	17 (85%)	22 (92%)	49 (98%)
Non-Han	3 (15%)	2 (8%)	1 (2%)
Organ involvement at diagnosis			
Liver	17 (85%)	–	–
Spleen	9 (45%)	–	–
Haematopoietic system	9 (45%)	–	–
Pituitary	1 (5%)	7 (29%)	–^b^
Lungs	12 (60%)	5 (21%)	–

^a^ One patient with mixed LCH and ECD.

^b^ One SS-LCH patient had reactivation with central diabetes insipidus during chemotherapy.

### Molecular features

**Figure 3.**
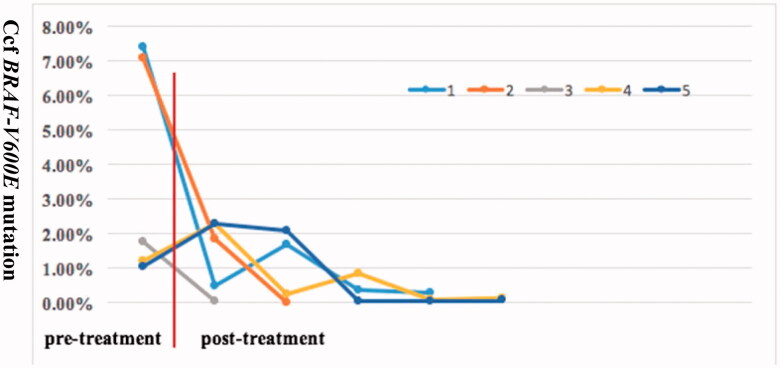
The result of ccf *BRAF-V600E* mutation load in five patients before therapy and after treatment. The treatment contained chemotherapy or/and vemurafenib. The quantitative detection of the ccf *BRAF-V600E* mutation was analysed by droplet-digital PCR assay. ccf: circulating cell-free.

The *BRAF-V600E* mutation was detected in 48 out of 84 patients who underwent gene analysis. Moreover, *N486_T491 > K*, *L485_P490delinsF* and *R506_K507insLLR*mutations in *BRAF* were identified in three cases without the *BRAF-V600E* mutation. Additionally, through next-generation sequencing, *ARAFp.Q349_F351delinsL* and *MAP2K1 p.Q58_E62del* were found in two patients. The *BRAF-V600E* mutation in biopsy tissue and peripheral blood were detected in 16 patients prior to therapy (Table 2). The concordance of *BRAF-V600E* mutation in tissue and blood was only 37.5% (6/16). Changes in *BRAF-V600E* mutation in blood were dynamically monitored in five patients during treatment (Figure 3).

**Table 2.** The result of *BRAF-V600E* genotype in the tissue and plasma prior to therapy in 16 patients.

	Tissue with *BRAF-V600E* mutation	Tissue without *BRAF-V600E* mutation
Plasma with *BRAF-V600E* mutation	5 patients	1 patient
Plasma without *BRAF-V600E* mutation	9 patients	1 patient

### Outcomes and treatment

The involved organ of the 50 SS-LCH patients in our study was as follows: bone, 48; skin, 1; lymph node, 1. The 48 bone-SS-LCH patients included 24 cases with multifocal lesions, 13 cases with special site unifocal lesion and 11 cases with non-special site unifocal lesion. Totally, modified LCH-III-based-protocol systemic chemotherapy was given to 46 SS-LCH patients (24 cases with multifocal bone lesions, 13 cases with special site unifocal bone lesions, eight cases with non-special site unifocal bone lesions and 1case with skin lesions). Four patients (three cases with non-special site unifocal bone lesions and 1case with lymph node) chose observation without chemotherapy due to NAD after surgery curettage. The above two patients with disease reactivation, switched to modified LCH-III-based-protocol chemotherapy, while the other two patients still had watch and wait procedure. Finally, 93, 11 and 5 patients received modified LCH-III-based-protocol chemotherapy, JLSG-02 protocol chemotherapy and *BRAF-V600E* inhibitor (vemurafenib), respectively. In the entire cohort, no patient received cladribine, high-dose cytarabine, clofarabine, allogeneic HSCT or radiotherapy.

In our study, one patient died because of severe encephalitis during maintenance chemotherapy, and 13 patients experienced disease reactivation (Table 3). In our study, two patients with disease reactivation were successfully controlled by modified-LCH-III protocol chemotherapy, while JLSG-02 protocol chemotherapy alone or in combination with vemurafenib salvaged the remaining 11 patients with refractory to modified-LCH-III protocol chemotherapy without severe toxic effects. Moreover, five patients who received vemurafenib therapy still could not stop this drug because of uncertainty of optimum treatment time, and the longest duration was 1 year and 1 month.

**Table 3.** Characteristics of 13 patients with disease reactivation.

	Numbers of patients
Status of disease	
Relapse	5
Progression	8
Clinical classification group	
SS-LCH	6^a^
RO − MS-LCH	3
RO + MS-LCH	4
The times of reactivation	
Once	11
Twice	2
Treatment strategy	
LCH-III based chemotherapy	5
JLSG-02 based protocol chemotherapy	11
Vemurafenib	5

^a^ Two SS-LCH patients relapsed without chemotherapy.

**Figure 4.**
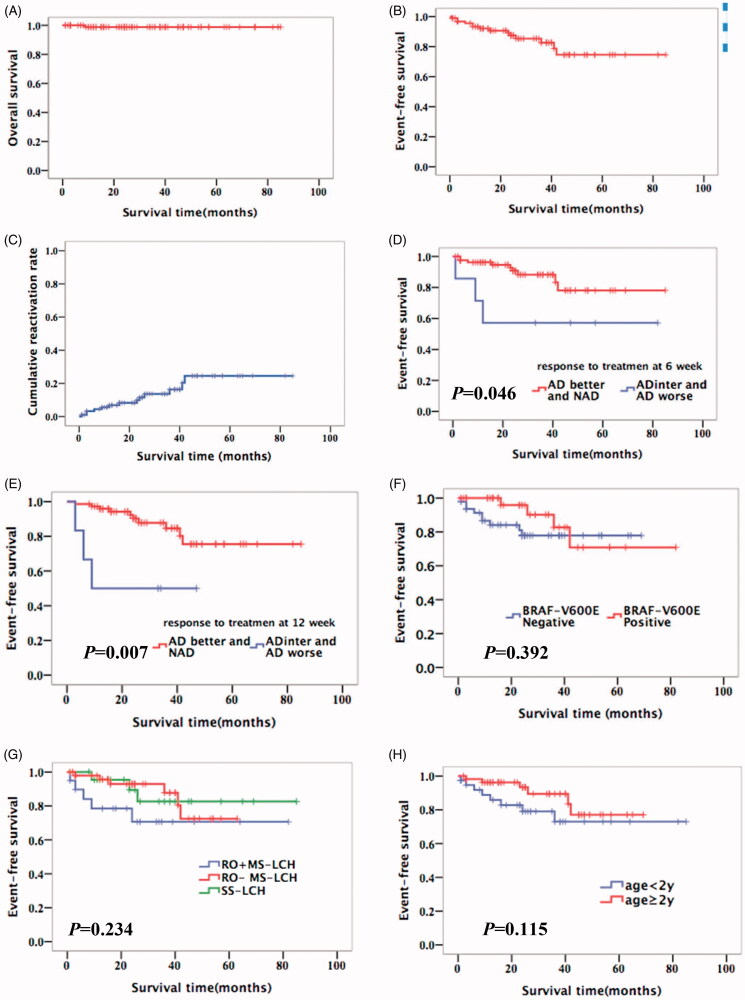
Kaplan–Meier’s curves for overall survival of the cohort (A), event-free survival of the cohort (B), cumulative reactivation of the cohort (C), patients according to different response to treatment at 6 weeks (D), patients according to different response to treatment at 12 week (E), patients according to*BRAF-V600E* (F), patients according to clinical classification (G) and patients according to age (H). NAD: non-active disease; AD: active disease; inter: intermediate.

The estimated five-year OS, EFS and cumulative reactivation rates of 95 patients in this study were 98.8% (95%CI, 97.6–100), 74.6% (95%CI, 67.6–81.6) and 24.5% (95%CI, 17.5–31.5), respectively (Figure 4(A–C)). According to modified LCH-III-based-protocol chemotherapy, 83 patients were NAD or AD/better, three patients were AD/intermediate and five patients were AD/worse after 6-week induction chemotherapy. Two patients did not reach the 6-week evaluation time point. Eighty patients received second 6-week induction chemotherapy and their response rate (NAD or AD/better) at 12-week was 94% (75/80). Three patients were AD/intermediate and two were AD/worse after 12-week induction chemotherapy. The five-year EFS rate in good responders was better than that in poor responders at 6-week (78% vs. 57%, *p* = .046) and at 12-week (75% vs. 50%, *p* = .007) (Figure 4(D,E)), while EFS was not affected by *BRAF-V600E* mutation, RO involvement or age (Figure 4(F–H)). However, EFS rates between good and poor responders were different only at 12-week according to the Cox proportional hazards model (HR = 0.022, 95%CI 0.002–0.231, *p* = .002).

### Sequelae

At diagnosis, central diabetes insipidus (CDI) was diagnosed in one RO + MS-LCH patient and seven RO − MS-LCH patients; after treatment, it was developed in another patient and was unresolved in them. Two patients showed growth retardation. No patients developed degenerative central nervous system disease, organ dysfunction or secondary tumours.

## Discussion

This is one of the largest single-institutional retrospective studies investigating the clinical characteristics and outcomes of children with LCH in China. Our study showed that the onset age of RO + MS-LCH patients was younger than that of RO − MS-LCH or SS-LCH, and that bone was the most frequently involved organ. More than 50% of the patients have *BRAF-V600E* mutation; however, *BRAF p.N486_T491 > K*, *BRAF p. L485_P490delinsF*, *BRAF p.R506_K507insLLR*, *MAP2K1 p.Q58_E62del* and *ARAF p.Q349_F351delinsL* mutations were also identified in the present study. According to modified LCH-III-based-protocol chemotherapy, JLSG-02 protocol chemotherapy and vemurafenib, the five year OS and EFS rates for the entire cohort were 98.8% and 74.6% and the five year cumulative reactivation rates were still high (24.5%), which were equivalent to that of developed countries [5,6]. Survival analysis showed that response rates (NAD and AD better) at initial therapy were significantly associated with better EFS, while age, RO involvement or *BRAF-V600E* mutation did not impact EFS in our study.

Langerhans cell histiocytosis is a rare disease, and until now, the national registry of childhood LCH in China is still not established, so the accurate incidence rate of children with LCH in China is unknown. As the largest paediatric oncology centre in Sichuan Province, we admit about 15 paediatric LCH cases every year. The ethnic distribution of the 95 patients in our study indicated that LCH was prevalent in many nationalities in China. The United States Registry studies found an increased incidence of LCH among Hispanics and a decreased incidence among African American children [12,13]. In our study, 85% of RO + MS-LCH patients were younger than two years of age. Similarly, large sample clinical studies from developed countries also suggested that the median age of diagnosis of RO + MS-LCH was less than that of RO– MS-LCH [12,13]. In fact, it is the state of precursor cell differentiation in which pathologic ERK activation arises and not the onset age that determines the clinical extent of LCH [14]. Therefore, we infer the earlier state of precursor cell differentiation in which pathologic ERK activation occurs, and a younger age of onset may be present. Although LCH may affect any organ, our study confirms that bone is the most frequently involved organ, followed by the skin, which is consistent with the guidelines of childhood LCH [15]. Hence, we conclude that if children present osteolytic lesions, especially with a seborrhoeic dermatitis like or an eczematous erythematous rash, LCH should be considered first.

In our centre, about 57% of Chinese paediatric LCH patients carried the *BRAF-V600E* mutation, which agrees with Badalian-Very et al.’s findings that more than 50% of LCH lesions from America had *BRAF-V600E* mutation [16]. In addition to *BRAF-V600E*, we found two deletion insertions (*BRAF p.N486_T491 > K*, *BRAF p. L485_P490delinsF*) and one insertion mutation (*BRAF p.R506_K507insLLR*) in *BRAF* from three *BRAF-V600E*-negative cases. Deletion of *MAP2K1* and deletion-insertion in *ARAF* was also identified in two cases. These findings further support the idea that 75% of LCH cases have mutually exclusive *MAPK* pathway-activating mutations, which are attributed to pathogenic LCH cells [4,17]. Recent evidence suggests that ccf *BRAF-V600E* mutational analysis in plasma provides a convenient and reliable method for detecting mutational status and is a promising non-invasive biomarker for monitoring the response to therapy in children with LCH [18,19]. However, in our study, the concordance between tissue and plasma ccf *DNA* genotype prior to therapy was only 37.5%, but ccf *BRAF-V600E* load at diagnosis significantly decreased with chemotherapy or and/or vemurafenib and remained at a low level during follow-up, which is consistent with a good clinical response. Based on our experience, monitoring ccf *BRAF-V600E* can serve as a method to evaluate the response to therapy, withdrawal time and disease reactivation, but it cannot replace the detection of *BRAF-V600E* in tissue.

The five-year OS rate in our study was 98.8%, the five-year EFS rate was only 74.6%. In our study, two of five SS-LCH patients with NAD after surgery relapsed without chemotherapy, but they still had a good response to modified LCH-III-based-protocol chemotherapy; therefore, we recommend that observation should be selected in unifocal SS-LCH with NAD after local therapy and full assessment. For MS-LCH, multifocal SS-LCH and special single-site SS-LCH, vincristine was used to replace vinblastine due to the lack of vinblastine in our hospital. Moreover, RO − MS-LCH patients received 12-month treatment and RO + MS-LCH patients accepted 18-month to 24-month treatment based on disease status without methotrexate. Additionally, multifocal SS-LCH and special single-site SS-LCH were treated with vincristine and prednisone for 6 months. Fortunately, results revealed that only 11 of 93 patients were refractory to vincristine and prednisone, and our study indicated that vincristine seemed to have the same effect on LCH without more adverse events compared with vinblastine. Moreover, it is somewhat surprising that RO involvement was not associated with poor survival, which differs from published studies [5,6]. A possible explanation for this might be that the therapy prolongation in RO + MS-LCH might improve outcomes. Another possible explanation for this is that partial RO + MS-LCH patients who responded poorly to chemotherapy in the early years were lost to follow-up. In our study, the results suggested that rapid responders had a better five-year EFS than poor responders, further supporting the idea of initial treatment response associated with prognosis in LCH.

To date, salvage treatment with MS-LCH refractory to vinblastine and steroid regimen is intractable. Nucleoside analogues may be a reasonable class of drugs for refractory LCH based on the hypothesis that LCH precursor cells are similar to myeloid precursors in cancers such as acute myeloid leukaemia. The combination of cladribine and cytarabine is an effective therapy for refractory LCH but is associated with a high treatment-related death rate [8,20]. Contrastingly, cytarabine or clofarabine monotherapy with a moderate dose in institutional research showed promising results without aggressive adverse events [10,21]. Although allogeneic HSCT may also be curative for refractory LCH with a three-year OS rate of 71–77%, the optimal choice of HSCT conditioning remains uncertain [9]. Vemurafenib is safe and effective in children with refractory *BRAF-V600E*-positive LCH, but the disease always reactivates with the withdrawal of vemurafenib [11]. As cladribine and clofarabine are unavailable in our hospital and we did not develop HSCT in refractory LCH, we chose JLSG-02 protocol chemotherapy and vemurafenib as salvage therapy for refractory MS-LCH. We found that JLSG-02 protocol chemotherapy alone or in combination with vemurafenib salvaged the 11 patients with refractory to modified-LCH-III protocol chemotherapy without severe toxic effects. However, five patients who received vemurafenib therapy still could not stop this drug and the longest duration was 1 year and 1 month. This unexpected finding suggests that JLSG-02 protocol chemotherapy and vemurafenib may be a feasible approach to refractory LCH. Finally, no serious sequelae, including degenerative CNS diseases, were found in our retrospective study.

Our retrospective study described the clinical features of children with LCH in China and displayed molecular traits in Chinese LCH. We found that prolongation therapy with vincristine and prednisone regimens could cure the majority of children with LCH. The JLSG-02 protocol chemotherapy and vemurafenib are promising candidates for refractory LCH. However, several limitations to this pilot study need to be acknowledged. The current research was only a retrospective study from a single centre in China without a large sample size, and further prospective randomized controlled clinical trials from multicentre research should be established in China to determine the optimal treatment protocol of LCH. Besides, the sample was nationally representative of LCH in our hospital but would tend to miss people who were lost to follow up. Finally, long-term safety of vemurafenib needs to further investigation, because the longest observation is just 1 year and 1 month in our study.

## Conclusions

We confirmed that LCH is a highly heterogeneous disease characterized molecularly by mutually exclusive *MAPK*-pathway-activating mutations. Based on our experience, poor initial treatment responses at 12-week significantly associated with poor survival. Vincristine, prednisone and cytarabine-based chemotherapy combined with vemurafenib improved the prognosis of childhood LCH. However, prospective clinical trials should be conducted to confirm our findings, and novel therapeutic strategies should be developed to improve outcomes in paediatric patients with LCH.

## Data Availability

Data available on request from the Dr Ju Gao.
